# A Potential Mechanism of High-Dose Ticagrelor in Modulating Platelet Activity and Atherosclerosis Mediated by Thymic Stromal Lymphopoietin Receptor

**DOI:** 10.1371/journal.pone.0141464

**Published:** 2015-10-30

**Authors:** Yi Mao, Yudong Peng, Qiutang Zeng, Longxian Cheng, Boyuan Wang, Xiaobo Mao, Kai Meng, Yuzhou Liu, Yitian Lian, Dazhu Li

**Affiliations:** Department of Institute of Cardiovascular Diseases, Union Hospital, Tongji Medical College, Huazhong University of Science and Technology, Wuhan, 430022, Hubei Province, PR China; University of Milan, ITALY

## Abstract

Abnormal expression of thymic stromal lymphopoietin (TSLP) and its receptor (TSLPR) was found in patients with acute coronary syndrome. Ticagrelor, an oral platelet ADP P2Y12 receptor antagonist, is widely used in these patients. The aim of this study was to verify whether different doses of ticagrelor regulated plaque progression and platelet activity by modulating TSLP/TSLPR. Seventy-five ApoE-/- mice were randomly divided into five groups: (1) high-cholesterol diet (HCD, n = 15); (2) HCD plus ticagrelor 25 mg/kg/d (T1, n = 15); (3) HCD plus ticagrelor 50 mg/kg/d (T2, n = 15); (4) HCD plus ticagrelor 100 mg/kg/d (T3, n = 15); and (5) a normal diet group (ND, n = 15). At day 0 and at week 16, blood lipids and serum TSLP levels, expression of TSLPR, CD62, and CD63, platelet aggregation, platelet ATP release, PI3K/Akt signaling pathway, and plaque morphology were assessed. HCD increased TSLPR expression and atherosclerosis progression but high-dose ticagrelor (100 mg/kg) moderated this trend. TSLPR was positively correlated with Akt1, platelet aggregation, corrected plaque area, and vulnerability index in the T3 group (P<0.01). In conclusion, low-dose ticagrelor only inhibited platelet activity. Besides this inhibition, high-dose ticagrelor modulated platelet activity and atherosclerosis mediated by TSLPR, potentially through the PI3K/Akt signal pathway.

## Introduction

Atherosclerosis (AS) is a chronic inflammatory disorder. Mortality due to AS is mainly caused by cardiovascular and cerebrovascular events due to atherothrombosis [1–3]. Studies have shown that the expression of thymic stromal lymphopoietin (TSLP), an inflammatory cytokine induced by angiotensin II and ox-LDL [4], and the TSLP receptor (TSLPR) were overexpressed in human AS plaques [5]. Locally in the AS plaque, some invasive cells derived from the bone marrow (such as mast cells, lymphocytes, dendritic cells (DCs), and macrophages) express functional TSLPR on their surface [6]. Interestingly, TSLPR deficiency attenuates atherosclerotic lesion development in apolipoprotein E-knockout (ApoE KO) mice [7]. One of our previous studies found for the first time that TSLPR expression on platelets was markedly increased in patients with acute coronary syndrome (ACS), which promoted the activation of platelets through phosphatidylinositol 3-kinase (PI3K) and its downstream effector Akt (PI3K/Akt signaling pathway) [8]. Therefore, TSLPR may be a novel regulation receptor for controlling platelet function.

Besides their key role in thrombosis, platelets also cooperate with an integral part of inflammation [9]. Upon command, platelets upregulate pro-inflammatory adhesion molecules, and promote interactions with endothelial cells and circulating leukocytes [9, 10]. Therefore, we consider that TSLP/TSLPR play an important role in atherothrombosis and AS progression.

As a direct-acting, reversibly binding oral platelet ADP P2Y12 receptor antagonist, ticagrelor has the ability to decrease the incidence of cardiovascular mortality, myocardial infarction, or stroke compared to clopidogrel in patients with ACS also treated with aspirin in the PLATelet inhibition and patient Outcomes (PLATO) trial [11]. In recent years, several pharmacological effects of ticagrelor other than agonistic platelet warranted high attention from researchers. For example, its inhibition of vascular smooth muscle cells (VSMCs) proliferation and benefits in the prevention of intimal hyperplasia in a rabbit carotid anastomosis model [12], and anti-inflammatory effects of ticagrelor such as inhibition of platelet-leukocyte aggregates and CD40 ligand shedding derived from platelet granule contents release [9]. Since the ticagrelor dose used in these studies was much higher than the clinical dose, we assume that high-dose ticagrelor may inhibit TSLP/TSLPR signaling, alleviating AS progression and inhibiting platelet activity.

To test this hypothesis, we first established an AS model using ApoE KO mice to explore the impact of different doses of ticagrelor on plaque morphology and platelet activity, which are closely related with the progression of atherothrombosis [13, 14], and further studied the role of ticagrelor and TSLP/TSLPR in this process.

## Materials and Methods

### Establishment of AS model

Seventy-five C57BL/6 ApoE^-/-^ mice were purchased from Jackson Laboratory (Bar Harbor, ME, USA) and were randomly divided into five groups after 1 week of acclimation: (1) high-cholesterol diet (HCD, n = 15): 1% cholesterol and 5% lard (Alpha Biotechnologies, Wuhan, China) for 16 weeks; (2) HCD plus ticagrelor (AstraZeneca, London, UK) 25 mg/kg/d by gavage for 16 weeks (T1, n = 15); (3) HCD plus ticagrelor 50 mg/kg/d by gavage for 16 weeks (T2, n = 15); (4) HCD plus ticagrelor 100 mg/kg/d by gavage for 16 weeks (T3, n = 15); and (5) a normal diet group (ND, n = 15). The ND and HCD group received saline as control. All animals were allowed to drink water ad libitum and gained weight during the process of the study. Mice were bred and maintained in the animal center of Beijing University. All animal studies were approved by the animal study committee of Tongji Medical College of Huazhong University of Science and Technology following the principles of the experimental animal ethics.

At day 0 and at week 16, fractionated blood was collected from the caudal vein in conscious mice (0.1–0.2 ml each time) to measure blood lipids, serum TSLP levels, ATP release, platelet aggregation, and expression of TSLPR, CD62, and CD63.

### Biochemistry analysis

Serum total cholesterol (TC), triglycerides (TG), and high-density lipoprotein cholesterol (HDL-C) were determined by spectrophotometry. Low-density lipoprotein cholesterol (LDL-C) was calculated according to the Friedwald formula [15]. Serum TSLP concentration was detected by enzyme-linked immunosorbent assay (ELISA), as suggested by the manufacturer (eBioscience, San Diego, CA, USA).

### Flow cytometry

Blood samples were drawn into plastic tubes containing 3.8% sodium citrate and centrifuged at 180 g for 10 min to obtain platelet-rich plasma (PRP). Washed platelet (WP) suspensions were obtained by centrifugation (1,000 g, 10 min) of PRP in the presence of prostacyclin [PGI2 (prostaglandin I2)] (75 nM), and the platelets were then washed in Tyrode buffer (145 mM NaCl, 5 mM KCl, 0.5 mM Na2HPO4, 1 mM MgSO4, 10 mM HEPES, 5 mM glucose, pH 6.5). The WPs were subsequently resuspended in Tyrode buffer, and the number of platelets was adjusted to 3×10^8^ cells/ml. After depletion of leucocytes using a high-efficiency leuco reduction filter (Purecell PL, Pall Corporation, Port Washington, NY, USA), highly purified platelets were incubated with a PE-conjugated anti-TSLPR antibody (20 μg/ml) (R&D Systems, Minneapolis, MN, USA) for 20 min and fixed in 0.5% paraformaldehyde. The expression of cell-surface TSLPR was detected using flow cytometry (FCM). After staining with a PE-conjugated anti-CD62 antibody (Biolegend, San Diego, CA, USA) or PE-conjugated anti-CD63 antibody (Biolegend, San Diego, CA, USA), the platelets were analyzed by FCM. The mean fluorescence intensity (MFI) of CD62 and CD63 was detected, and the increase in MFI of platelets compared with that in control groups was recorded. Relative isotype controls were employed to enable correct compensation and confirm antibody specificity. Data were detected using a Becton Dickinson FACScan flow cytometer and analyzed using the CELLQUEST software (BD Biosciences, Franklin Lake, NJ, USA).

### Hematoxylin-eosin staining and Masson staining

Cross-sections of the proximal aorta just below the aortic valve ring were fixed in 10% buffered formalin solution for 24 hours, embedded in paraffin, and serial 5-μm sections were cut for immunohistochemistry, hematoxylin-eosin (HE) staining (Shanghai Ruiqi Technology, China), and modified Masson staining (Absin Bioscience, China). HE staining could clearly show the plaque area, but the plaque area was corrected by dividing the surrounding area of internal elastic lamina. The Masson staining protocol was used to produce red collagen and muscle, blue or green mucilage, achromatic foam cells and extracellular lipids, and dark brown to black cell nuclei. AS plaque is mainly composed of foam cells, extracellular lipids, and collagen, and the percentage of each component in the whole plaque was calculated. The vulnerability index was calculated as: vulnerability index = (extracellular lipids area + foam cell area)/(collagen area + SMC area) [16, 17]. To quantitatively evaluate AS lesions, approximately 100 serial cross sections (5-μm thick) of the ascending aorta were prepared according to the method described by Yao et al [18] and were used for analysis with an automated computerized image analyzer (ImagePro Plus, Media Cybernetics, Inc., Rockville, MD, USA). Descending aorta segments were snap-frozen in liquid nitrogen and stored at -70°C for western blot analysis and real-time PCR.

### Immunohistochemistry

Serial 5-μm paraffin sections were dewaxed and rehydrated. Endogenous peroxidase activity was inhibited by incubation with 3% hydrogen peroxide. After blocking sections with 20% (v/v) goat serum in phosphate-buffered saline, sections were incubated overnight at 4°C with TSLPR antibody (1:200; R&D Systems, Minneapolis, MN, USA) and Akt1 antibody (1:500; Santa Cruz Biotechnology, Santa Cruz, CA, USA). Sections were then incubated with the appropriate secondary antibodies. Blinded analysis of positive immunostained sections was performed with the image-analysis program (ImagePro Plus, Media Cybernetics, Inc., Rockville, MD, USA).

### Platelet aggregation and ATP release

Platelet aggregation and ATP release were measured in a lumi-aggregometer (Chrono-Log Corp., Havertown, PA, USA). Ca^2+^ (1 mM) was added to the WPs, which were then incubated separately with collagen (5 μg/ml) (Chrono-Log Corp., Havertown, PA, USA). Platelet aggregability was evaluated according to the maximal percentage of platelet aggregation [19, 20]. ATP levels were measured at the end of the assay by adding a known amount of an ATP standard (2 μM) [19, 20]. Curves were analyzed using the HemoRAM software.

### Real-Time Reverse-Transcription PCR

Total RNA was harvested from mice aortas. Following the aggregation reaction, total RNA of platelets was prepared using Trizol Plus (Takara Bio, Otsu, Japan). cDNA was transcribed from purified RNA using a RNA PCR Kit (Takara Bio, Otsu, Japan). Real-time polymerase chain reaction (PCR) was performed using the One Step SYBR Green Mix (Takara Bio, Otsu, Japan) and an ABI Prism 7900 Sequence Detection System (Applied Biosystems, Foster City, CA, USA) according to the manufacturers’ instructions. Melting curves were used to determine the abundance of the transcripts after 40 cycles of 30 seconds at 94°C, 30 seconds at 57°C, and 30 seconds at 72°C. Amplification reactions were performed in duplicate, and mRNA expression was calculated using the comparative CT method formula 2^-ΔΔct^. Data were normalized to β-actin. Real-time reverse-transcription polymerase chain reaction (PCR) primers were:

TSLPR forward, 5’- CTT CGC AGG GTG AAA GAT GC -3’;

reverse, 5’- CCC TCT TAG CCT TGG TGT GG -3’.

Akt1 forward, 5’- CCG CCT GAT CAA GTT CTC CT -3’;

reverse, 5’- AGA GGG AGA GGG CCA GTT AG -3’.

β-actin forward, 5’- CCT CTA TGC CAA CAC AGT GC -3’;

reverse, 5’- CCT GCT TGC TGA TCC ACA TC-3’.

### Western blotting

Total proteins, cytoplasmic extracts, membranous extracts, and nuclear extracts were prepared from pooled arteries with RIPA lysis buffer (Beyotime Biotechnology, Haimen, China). The concentrations of proteins were determined using the BCA protein assay (Pierce Chemical, Dallas, TX, USA). Following the aggregation reaction, the platelets were centrifuged. The precipitated platelets were washed twice with PBS and then lysed in lysis buffer. The lysates were centrifuged and denatured for 10 minutes. The protein samples were subsequently separated using SDS-PAGE and analyzed by western blotting. The primary antibodies included TSLPR antibody (1:200; R&D Systems, Minneapolis, MN, USA), phosphorylated Akt1 (p-Akt1) antibody (1:500; Santa Cruz Biotechnology, Santa Cruz, CA, USA) and a GAPDH antibody (1:1000; Sigma, St Louis, MO, USA). Nitrocellulose membranes were used for protein transfer. A chemiluminescence reagent (Thermo Fisher Scientific, Waltham, MA, USA) was used to detect the chemiluminescent signal.

### Statistical analysis

Values are shown as means ± SEM in each group, and all data were analyzed using SPSS 19.0 (IBM, Armonk, NY, USA). Differences were evaluated by one-way analysis of variance (ANOVA) with the Newman-Keuls post hoc analysis. Pearson’s correlation was used to test for correlations between two continuous variables. A P-value <0.05 was considered to be significant.

## Results

### Biochemical indexes

No significant difference in body weight was observed among groups. At baseline, all indices showed no significant difference between groups (P>0.05, Table 1). After 16 weeks of HCD feeding, blood lipids of all groups were higher than the ND group (all P<0.05), but there was no difference between the four HCD groups (all P>0.05). Ticagrelor did not affect blood lipids and serum TSLP levels (Table 2).

**Table 1:** Blood lipids, serum TSLP and weight levels in the five groups at the baseline.

	HCD	T1	T2	T3	ND
	(n = 15)	(n = 15)	(n = 15)	(n = 15)	(n = 15)
TC (mmol/L)	3.14±0.29	3.11±0.28	3.15±0.24	3.04±0.28	3.15±0.33
TG (mmol/L)	1.73±0.14	1.75±0.15	1.76±0.20	1.77±0.17	1.75±0.13
LDL (mmol/L)	2.85±0.16	2.88±0.17	2.89±0.21	2.85±0.17	2.84±0.14
TSLP (μg/L)	5.63 ±0.19	5.65 ±0.16	5.61±0.20	5.64±0.2	5.64 ±0.16
Weight at baseline (g)	18.95±0.90	18.75±0.85	18.59±1.00	18.74±0.72	18.29±0.58

ND, HCD, T1, T2 and T3 groups at the baseline. TC: total cholesterol; TG: triglyceride; HDL-C: high-density lipoprotein cholesterol; LDL-C: low-density lipoprotein cholesterol. TSLP: Thymic stromal lymphopoietin.

**Table 2:** Blood lipids, serum TSLP and weight levels in the five groups at the week 16.

	HCD	T1	T2	T3	ND
	(n = 15)	(n = 15)	(n = 15)	(n = 15)	(n = 15)
TC (mmol/L)	23.31±1.39	22.79±1.58	23.95±1.44	23.65±1.88	3.91±0.39
TG (mmol/L)	3.61±0.98	3.7±0.9	3.57±0.86	3.17±1.35	2.29±0.5
LDL (mmol/L)	21.72±4.9	21.56±3.8	21.37±5.94	21.33±3.55	3.15±1.34
TSLP (μg/L)	8.34 ±2.85	8.63 ±2.5	7.56±2.81	7.99±2.1	4.72 ±1.56
Weight at 16 weeks (g)	28.41±1.09	28.16±1.28	28.55±2.02	28.11±1.69	28.19±2.06

ND, HCD, T1, T2 and T3 groups were treated as described in the Methods section. TC: total cholesterol; TG: triglyceride; HDL-C: high-density lipoprotein cholesterol; LDL-C: low-density lipoprotein cholesterol. TSLP: Thymic stromal lymphopoietin.

### Expression of TSLPR and platelet surface molecules

**Fig 1:**
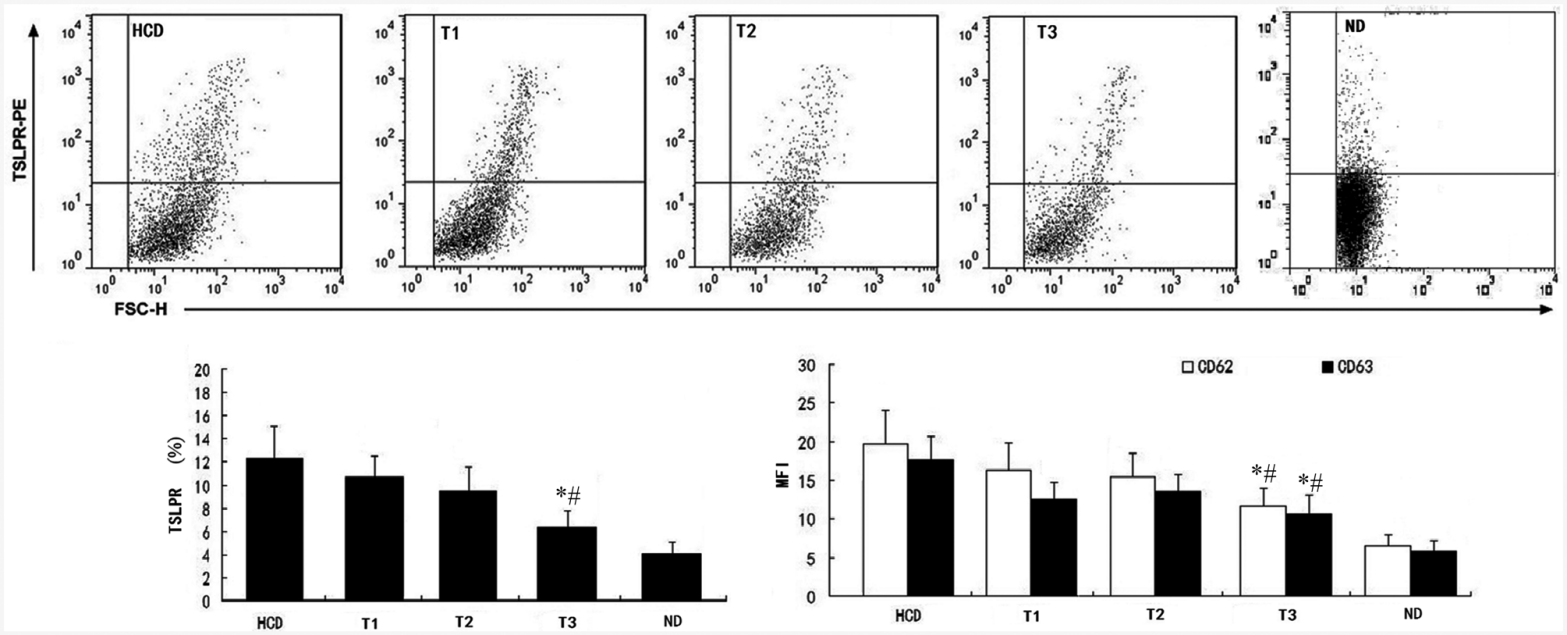
After 16 weeks of high-cholesterol diet, TSLPR, CD62, and CD63 expression on platelet surface were not significantly different between the HCD, T1 (ticagrelor 25 mg/kg/d), and T2 (ticagrelor 50 mg/kg/d) groups. Ticagrelor at 100 mg/kg/d in the T3 group significantly decreased TSLPR, CD62, and CD63 expression on platelet surface. *P<0.01 vs. HCD. #P<0.01 vs. T1 and T2.

Expression of TSLPR, CD62, and CD63 in the T1 and T2 groups was not decreased compared to the HCD group (*P*>0.05). However, ticagrelor at 100 mg/kg/d significantly decreased TSLPR, CD62, and CD63 expression in the T3 group compared with that of the other three groups (all P<0.01) (Fig 1).

### Platelet activity detection

**Fig 2:**
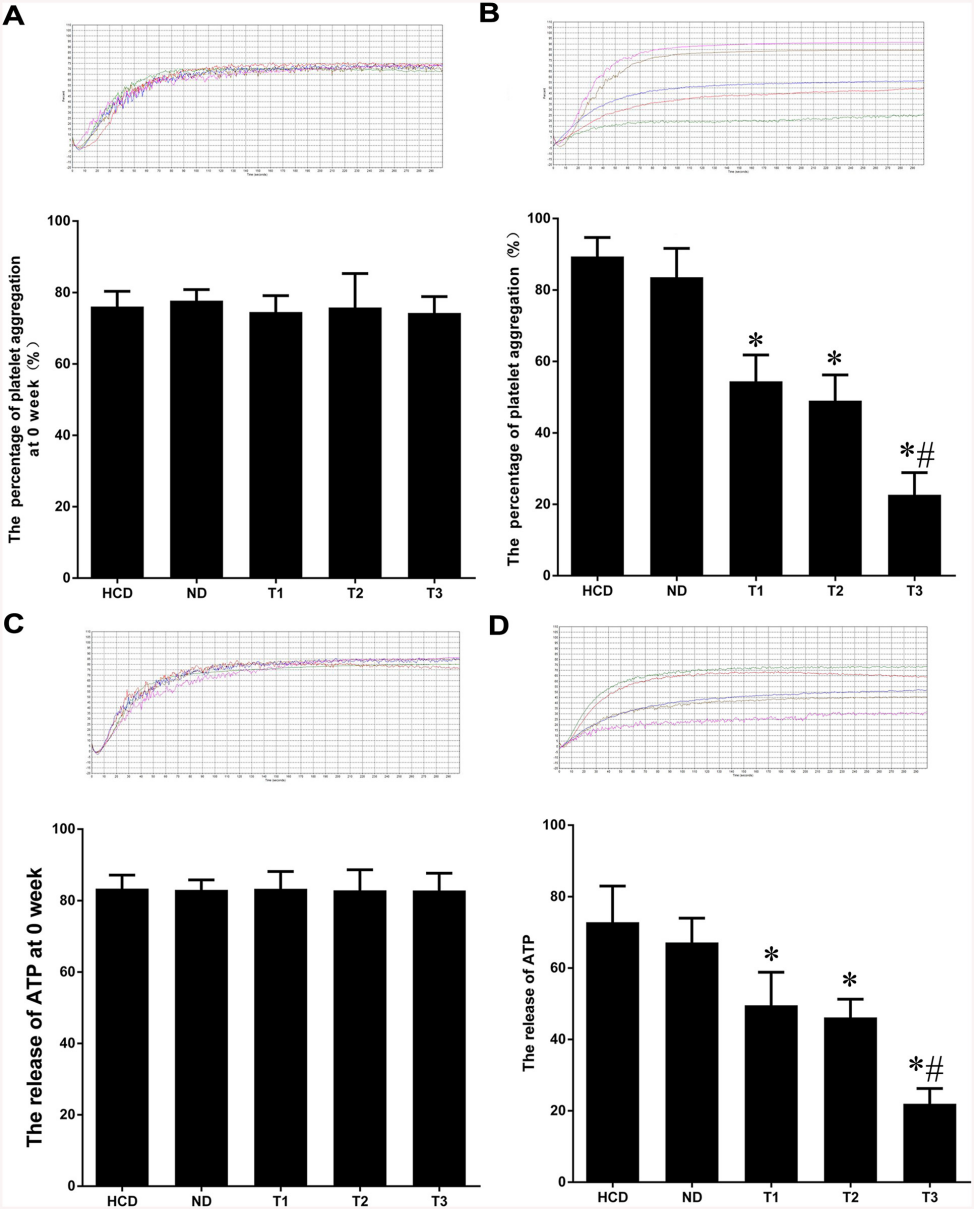
Representative experiments for platelet aggregation (A, 0 weeks; B, 16 weeks) and ATP release (C, 0 weeks; D, 16 weeks). At baseline, platelet activity showed no significant difference between groups. After 16 weeks, the maximal percentage of platelet aggregation and ATP release between each ticagrelor group and HCD group induced by collagen (5 μg/ml) was different (P<0.01), but there was no difference between the T1 (ticagrelor 25 mg/kg/d) and T2 (ticagrelor 50 mg/kg/d) groups. *P<0.01 vs. HCD. #P<0.01 vs. T1 and T2.

At baseline, platelet activity showed no significant difference between groups (Fig 2A and 2C). After 16 weeks, the maximal percentage of platelet aggregation was 88.07±4.97% in the HCD group, 54.37±6.1% in the T1 group, 49.11±5.9% in the T2 group, and 22.7±5.1% in the T3 group. The concentration of ATP was 72.81±8.2 μM in the HCD group, 49.48±7.56 μM in the T1 group, 46.22±4.17μM in the T2 group, and 22.04±3.59 μM in the T3 group. There were no differences between the HCD and ND groups and no differences between the T1 and T2 group, but the T1 and T2 groups were different from HCD/ND (P<0.01). The aggregation and ATP release of platelet for T3 were different from T1 and T2 (P<0.01) (Fig 2B and 2D).

### Histomorphometric analysis

**Fig 3:**
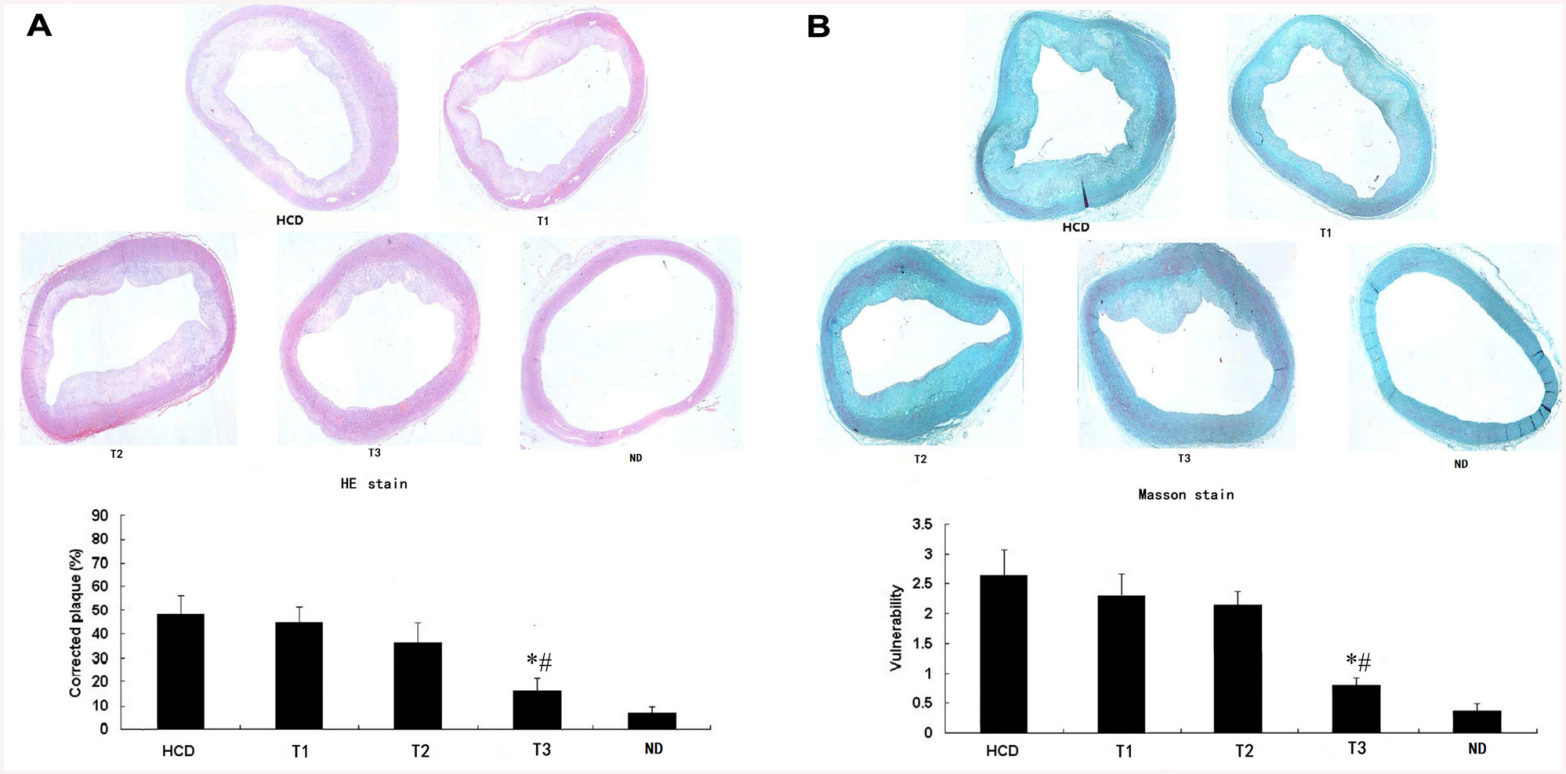
Photomicrographs of representative sections of ascending aorta from the five groups (magnification 40×). After 16 weeks of high-cholesterol diet, larger corrected plaque area and vulnerability index were observed in the HCD, T1, and T2 groups compared with those in the T3 group. However, there was no significant difference of corrected plaque area and vulnerability index between the HCD, T1 and T2 groups. *P<0.01 vs. HCD. #P<0.01 vs. T1 and T2.

**Fig 4:**
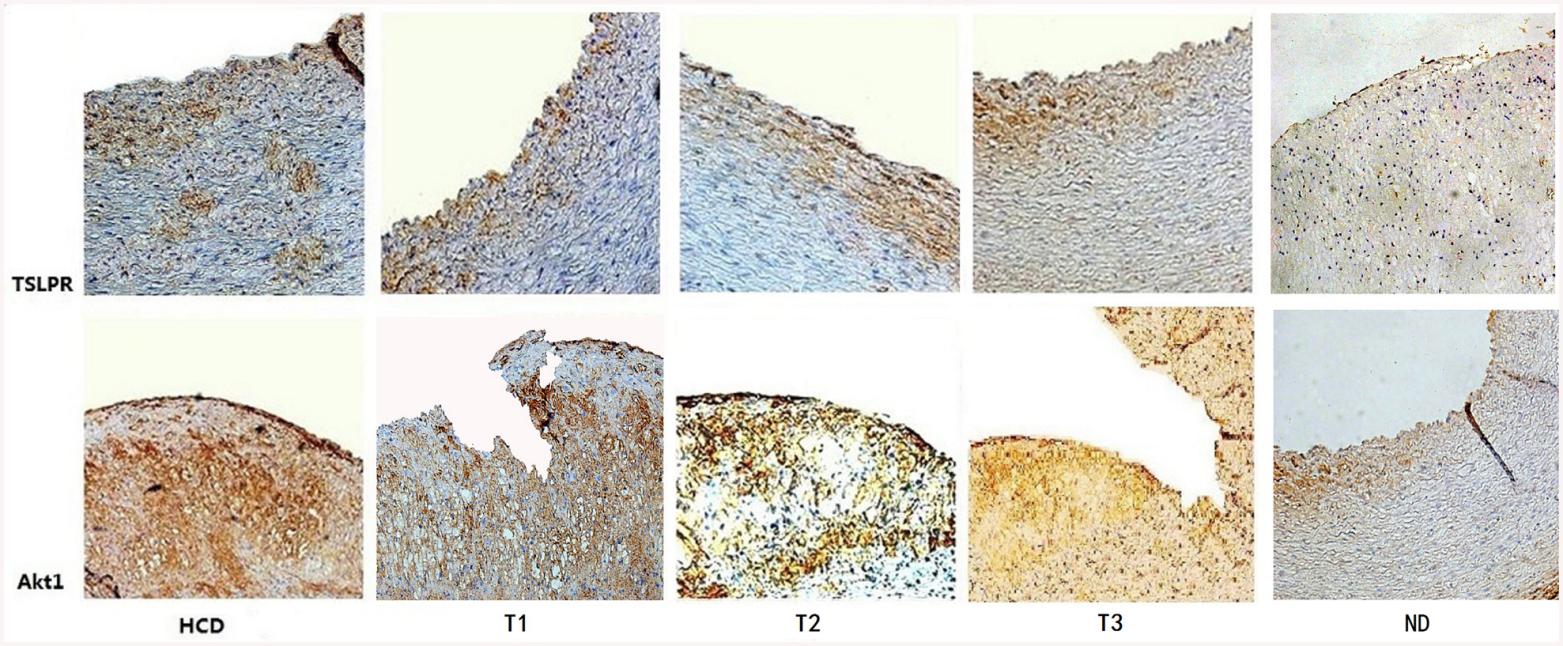
Photomicrographs of representative sections of ascending aorta from the five groups (immunohistochemistry, 200×). Vessel wall cells stained dark blue were considered positive for TSLPR expression. Cytoplasm and matrix stained yellow brown were considered positive for Akt1 expression. The mean density of TSLPR coincided well with that of Akt1 in plaques. HCD increased the expression of these markers, while only high-dose ticagrelor (100 mg/kg/d) and ND decreased their expression.

After 16 weeks, larger corrected plaque area and vulnerability index were observed in the HCD, T1, and T2 groups compared to the T3 group (P<0.01). However, there were no significant differences in corrected plaque area and vulnerability index between the HCD, T1, and T2 groups (P>0.05). The ND group showed no significant AS (Fig 3). Vessel wall cells stained dark blue were considered positive for TSLPR expression. Cytoplasm and matrix stained yellow brown were considered positive for Akt1 expression. Immunohistochemistry showed that the mean density of TSLPR coincided with that of Akt1 in local plaques. HCD increased their expression, while only high-dose ticagrelor (100 mg/kg/d) decreased their expression. The ND group showed very low TSLPR and Akt1 expression (Fig 4).

### Effect of ticagrelor on signal-transduction pathway

**Fig 5:**
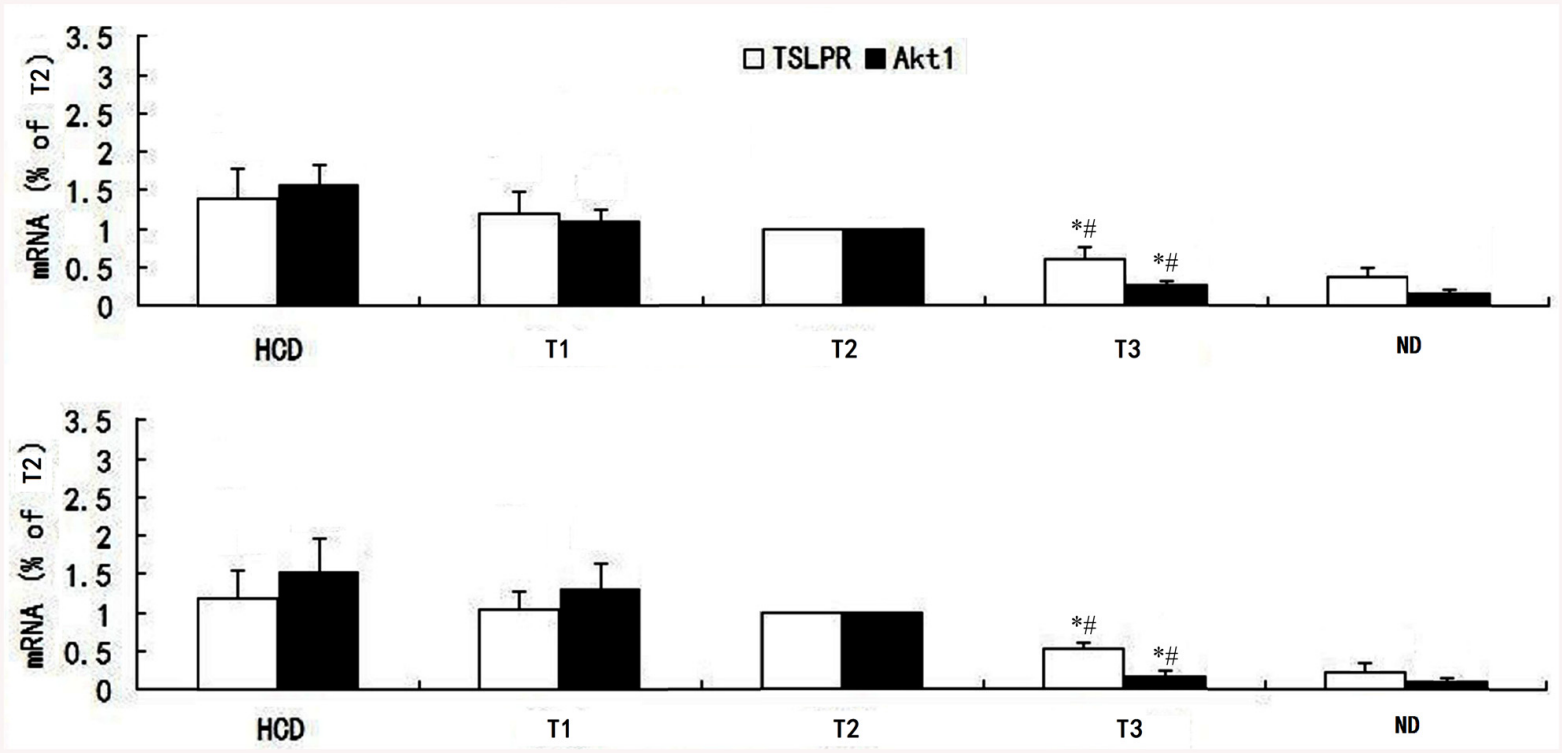
Gene expression of Akt1 and TSLPR (mRNA) in each group. Local vascular histology (top) and cytology platelet levels (bottom) showed higher levels of Akt1 and TSLPR in the HCD, T1, and T2 groups. The two indices were markedly decreased in the T3 group (100 mg/kg/d). There was no significant difference between the HCD, T1, and T2 groups. *P<0.01 vs. HCD. #P<0.01 vs. T1 and T2.

**Fig 6:**
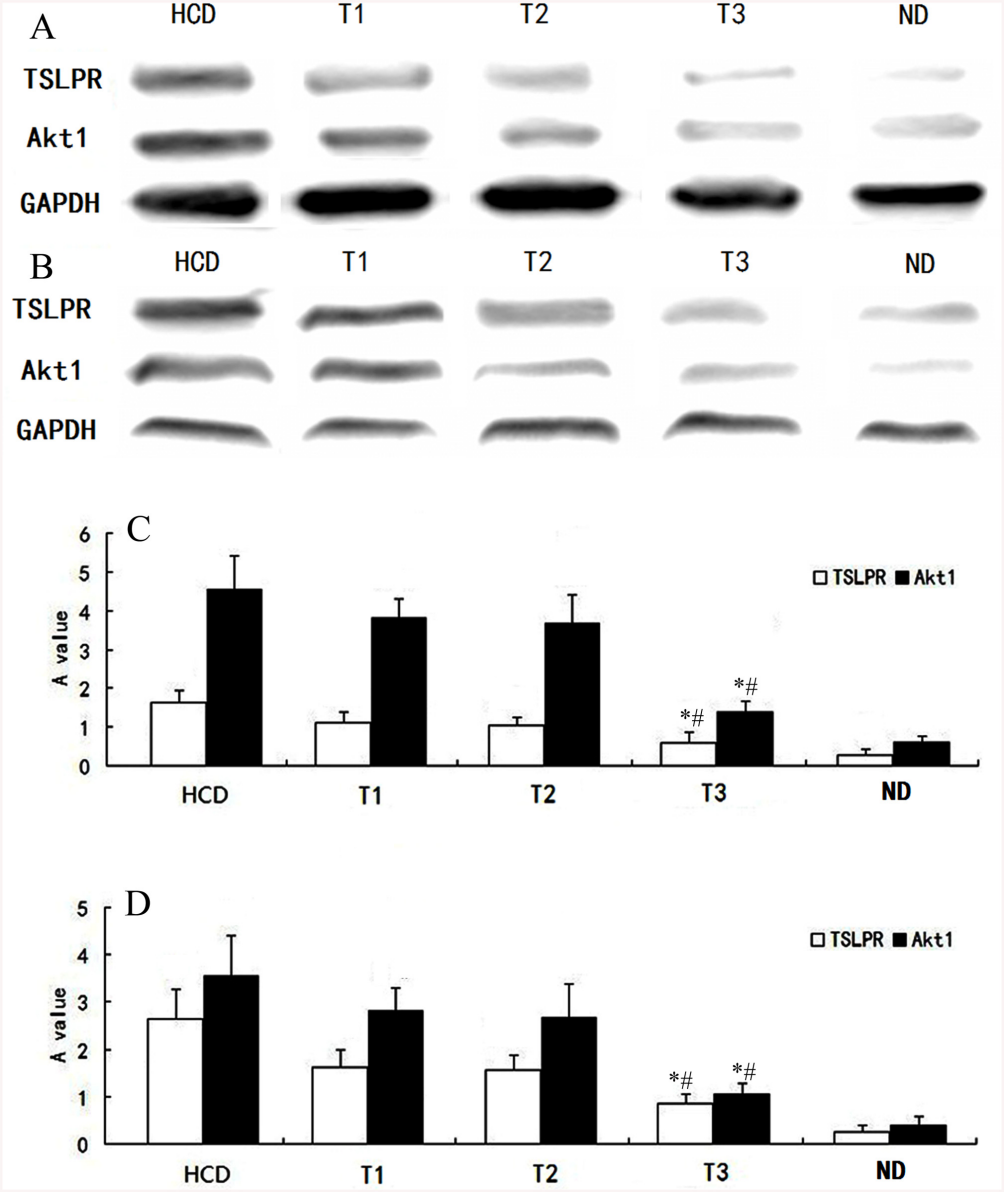
Representative western blots of TSLPR, Akt1, and GAPDH in all groups (A, B). Values are presented in the bottom histogram for the five groups (C, D). Vascular histology (A, C) and cytology platelet levels (B, D) showed higher levels of Akt1 and TSLPR in the HCD, T1, and T2 groups. *P<0.01 vs. HCD. #P<0.01 vs. T1 and T2.

From local vascular histology and cytology platelet levels, we measured the expression and transcription of TSLPR and Akt1 levels using RT-PCR and western blotting. As shown in Figs 5 and 6, PCR and western blotting revealed that HCD markedly enhanced TSLPR and Akt1 expression, and only high-dose ticagrelor (100 mg/kg/d) alleviated this effect.

### Correlations

**Fig 7:**
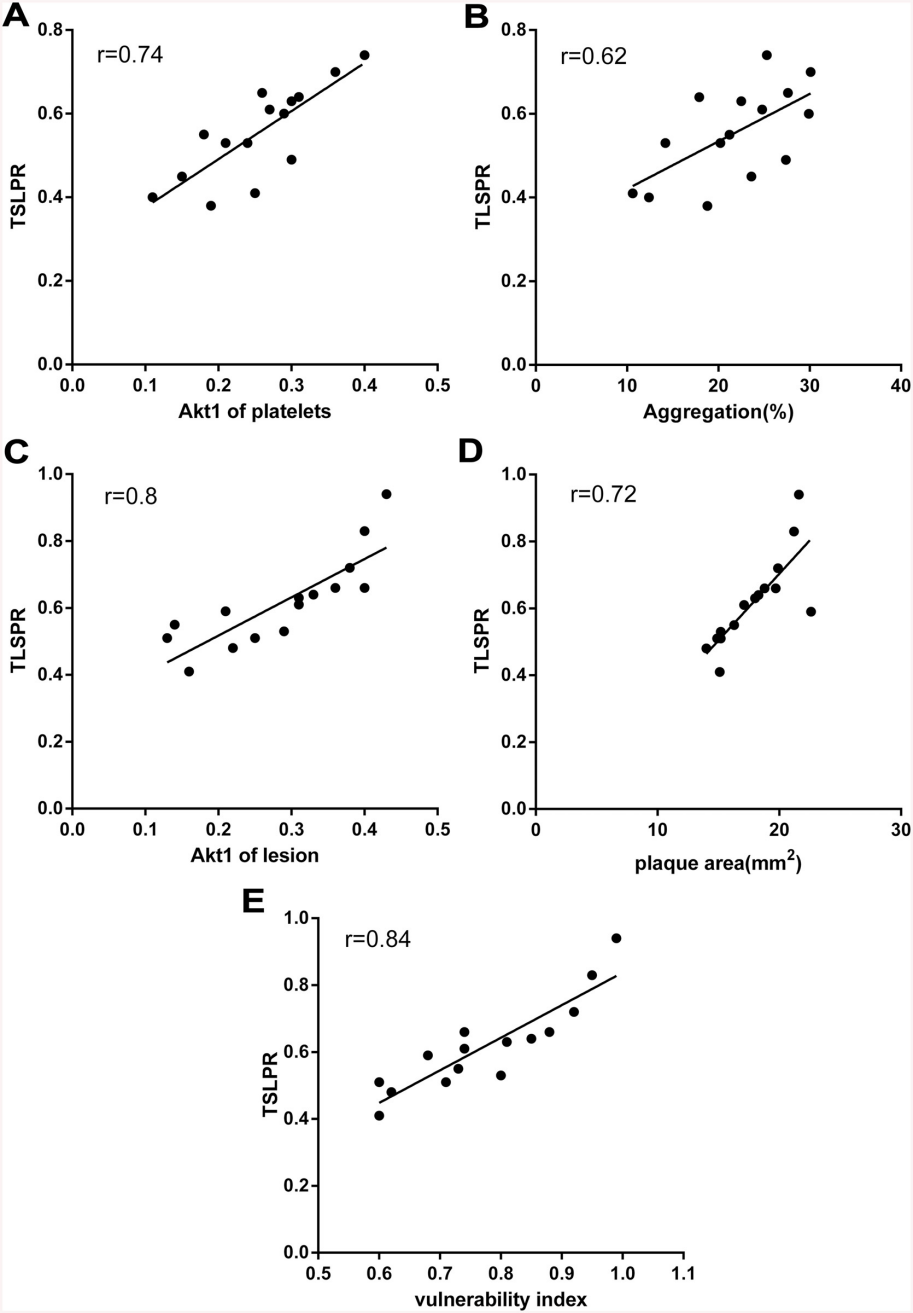
Correlation coefficients and the scatterplots of TSLPR in transcription with each index of T3 group. (A) Correlation between TSLPR transcriptional level from platelet (mRNA expression) and Akt1 mRNA expression from platelet. (B) Correlation between TSLPR transcriptional level from platelet (mRNA expression) and aggregation. (C) Correlation between TSLPR transcriptional level from vascular histology (mRNA expression) and Akt1 mRNA expression of plaque lesion. (D) Correlation between TSLPR transcriptional level from vascular histology (mRNA expression) and palque area. (E) Correlation between TSLPR transcriptional level from vascular histology (mRNA expression) and vulnerability index. P<0.01 for all.

At the transcription level, TSLPR on platelets was positively correlated with platelet Akt1 and platelet aggregation in the T3 group. Local TSLPR in lesions was positively correlated with lesion Akt1 and corrected plaque area as well as vulnerability index in the T3 group (Fig 7).

## Discussion

Plaque rupture and thrombosis are two important processes contributing to ACS occurrence. Plaque stability is determined by the composition of the AS plaque and the thickness of the fibrous cap [21]. Vulnerable plaques are fragile and can contribute to atherothrombosis, which is closely related to inflammatory factors [22]. TSLP is a recently discovered inflammatory cytokine that exerts biological effects through TSLPR [23]. Previous studies have shown that many types of myeloid and lymphoid cells express TSLPR such as monocytes, DCs, T-cells, and B-cells [8]. TSLP/TSLPR also promotes T lymphocyte-mediated inflammatory and immune injury. TSLPR is over-expressed in local rheumatoid arthritis lesions, bronchial asthma and atopic dermatitis, and is involved in the development of these diseases [20, 23, 24]. These diseases are often prone to be complicated by thrombotic events [25, 26]. In a previous study, we found that TSLPR was expressed on platelets, and that its expression was significantly increased in patients with ACS. TSLP/TSLPR functions via activating the PI3K/AKT pathway to promote platelet activation [8], and this signal pathway may be one of the mechanisms involved in plaque rupture and thrombosis in ACS.

As a relatively novel anti-platelet agent, ticagrelor has been shown to reversibly inhibit ADP-P2Y12 receptors on platelets, which is the so-called ADP-dependent platelet inhibition. The PLATO trial showed an overall reduction in all-cause and cardiovascular mortality with ticagrelor and a decrease in myocardial infarction and stent thrombosis compared with clopidogrel [27]. These results strongly suggest that this mortality benefit is only due to more effective ADP receptor inhibition. However, with the presentation of the latest results, other cardiovascular protective effects of ticagrelor are currently being explored. For instance, studies are focusing on its inhibition of SMC proliferation and benefits in the prevention of intimal hyperplasia [12], its improvements of coronary blood flow and myocardial perfusion in infarction by increasing cyclic adenosine concentration [10], and its anti-inflammatory effects such as inhibition of platelet-leukocyte aggregates and CD40 ligand shedding [9]. In addition, these cardiovascular protective effects are observed to be dose-dependent. In the present study, TSLPR and high-dose ticagrelor regulated platelet function and AS progression. Therefore, it is worth to further explore whether ticagrelor affects platelet function and plaque structure by regulating TSLPR expression.

Aggregation and coupling of activated platelets are the pathological basis of acute vascular thrombosis, which is closely associated with platelet membrane receptors and membrane surface molecule expression [2, 3]. In the present study, ticagrelor from 25 mg/kg/d to 100 mg/kg/d had not significant effect on blood lipids and serum TSLP concentration, and ticagrelor at 25 mg/kg/d (equivalent to the clinical routine therapeutic dose) and 50 mg/kg/d had no effect on platelet TSLPR and surface molecules CD62 and CD63 expression. However, ticagrelor at 100 mg/kg/d significantly reduced TSLPR and surface molecules CD62 and CD63 expression. CD62, also known as P-selectin, facilitates the formation of a platelet thrombus and induces platelet-leukocyte aggregation mediated by the interaction of activated platelets or endothelial cells with leukocytes. CD63 is a transmembrane protein and undergo a conformational change when platelets are activated, which could induce GPIIb/IIIa integrin and link to PAC-1 and expose a ligand-binding site for adhesive macromolecules including fibrinogen, von Willebrand factor, and fibronectin. Binding of fibrinogen to GPIIb/IIIa is required for platelet aggregation [28–30]. The increase of aggregation and ATP release are typical indicators of platelet activation [31]. As specific ADP receptor antagonist, low-dose ticagrelor only antagonize the ADP receptor, mildly inhibiting platelet aggregation and ATP release, and there was no significant difference between the 25 mg/kg/d and 50 mg/kg/d groups. Correlation analysis indicated that the expression of TSLPR was positively correlated with platelet activity in the T3 group. This strongly suggests that high dose ticagrelor can also inhibit TLSPR and membrane surface molecule expression besides inhibiting the ADP receptor pathway, more remarkably suppressing platelet aggregation and ATP release by other means such as the thromboxane A2 (TXA2) and platelet activating factor (TAF) pathway [28].

Increased plaque burden and plaque vulnerability are the most important pathogenic processes involved in plaque progression. Recently, Shiomi et al. have proposed the morphometric “vulnerability index”, which is the ratio of plaque area occupied by lipid components (macrophages and extracellular lipids) and by fibromuscular components (smooth muscle cells and collagen fibers) [17]. The present study demonstrated that the corrected plaque area and vulnerability index in the high-dose T3 group were obviously decreased, indicating that plaques were more stable. However, after being treated using low-dose ticagrelor in the T1 and T2 groups, the corrected plaque area and vulnerability index were not decreased markedly. The expression of TSLPR was positively correlated with plaque morphometric index in the T3 group. The potential mechanism implied that high-dose ticagrelor might play a new role on inhibiting plaque progression mediated by TSLPR.

Our previous study showed that TSLP/TSLPR functions via activating the PI3K/AKT pathway and that this signaling pathway might be one of the mechanisms involved in thrombosis in ACS [8]. Our additional research found that the concentration of TSLPR was positively correlated with Akt1 levels of platelet, that the expression of TSLPR on platelets was positively correlated with platelet activity in the T3 group, and that the local expression of TSLPR in lesions was positively correlated with Akt1 and plaque morphometric index in the T3 group. As a P2Y12 receptor antagonist, some studies focused on the use of ticagrelor for the prevention of intimal hyperplasia and its anti-inflammatory properties, beyond its anti-platelet aggregation effect [9–12]. However, the ticagrelor doses used in these studies were much higher than its clinical dose [9–12]. Therefore, ticagrelor might decrease cardiovascular mortality in patients with ACS by other mechanisms. For example, it is undeniable that statins decrease LDL-C, but their benefit is not entirely explained by their inhibition of the HMG-CoA reductase. Similarly, ticagrelor exerts its effects through rapid and consistent platelet inhibition, but these effects on platelet function and plaque composition are not entirely explained by the inhibition of the P2Y12 receptor. Different from ADP receptor, TSLPR expression present high reactivity in the emergency stage of ACS [23, 25, 26], and the complete inhibition of TSLPR might need a large dose of ticagrelor. Further study is necessary to address this issue.

## Conclusion

Low-dose ticagrelor only inhibited platelet activity, while high-dose ticagrelor inhibited platelet activity and AS mediated by TSLPR, probably through the PI3K/AKt signal pathway.

## Supporting Information

S1 Raw Data (XLS)
